# Thrombotic Thrombocytopenic Purpura Presenting as a Complex Migraine: A Case Report

**DOI:** 10.7759/cureus.78072

**Published:** 2025-01-27

**Authors:** Devon D Thorpe, Pragnesh Patel, Kiana Bhola, Aurora Cafuli, Paul Markowski

**Affiliations:** 1 Internal Medicine, Overlook Medical Center, Summit, USA; 2 Hematology and Oncology, Overlook Medical Center, Summit, USA

**Keywords:** adamts13, complex migraine, hematologic emergency, neurologic symptoms, plasmapheresis, thrombotic thrombocytopenic purpura

## Abstract

Thrombotic thrombocytopenic purpura (TTP) is a rare and life-threatening disorder characterized by microangiopathic hemolytic anemia, thrombocytopenia, fever, neurologic symptoms, and renal dysfunction. Neurologic manifestations can range from mild headaches to severe sequelae such as seizures, stroke, and coma. TTP may present atypically, including with symptoms such as complex migraines, which can complicate diagnosis and delay treatment. Early recognition and prompt treatment are essential, as untreated TTP carries a high mortality rate. This case report highlights the importance of considering TTP in the differential diagnosis of patients presenting with a first-time complex migraine, as it can occasionally be the initial manifestation of this rare and serious condition. It also underscores the necessity of performing a complete blood count (CBC) in patients with new neurological symptoms, as complex pathologies like TTP can present with overlapping features. We present the case of a 19-year-old male whose initial complaint of a complex migraine led to the diagnosis and successful treatment of TTP with plasmapheresis, corticosteroids, and rituximab.

## Introduction

Thrombotic thrombocytopenic purpura (TTP) is a rare life-threatening disease with an estimated annual incidence rate of 1.5-6 cases per one million adults annually worldwide. In the United States, African American people are disproportionately affected, accounting for the higher incidence in this population [1,2]. Classically, TTP presents as a pentad of microangiopathic hemolytic anemia (MAHA), thrombocytopenia, fever, neurologic abnormalities, and renal failure [3]. Neurologic symptoms - developing as headache, altered mental status, seizures, and focal deficits - are seen in approximately 60% of patients [4], but not all cases present with the full pentad. In some instances, neurologic signs may even precede the characteristic hematologic features and further confound diagnosis. TTP rarely presents an easy diagnosis, particularly because, in the majority of cases, neurologic symptoms prevail. Because of its rarity, diagnosis requires a high index of suspicion, particularly among patients with vague or nonspecific neurological complaints. ADAMTS13 activity is usually markedly low (<10%) in TTP; therefore, a delay in treatment should not be made in waiting for these results [5]. Early intervention with plasmapheresis, corticosteroids, and rituximab is associated with improved outcomes and lower mortality rates [6]. In this report, a differential diagnosis of TTP is to be entertained in a patient presenting with severe headache or neurologic symptoms where hematologic manifestations may be very subtle or even absent during the initial presentation.

## Case presentation

A 19-year-old male with no significant past medical history presented to the ED with a severe, new-onset, left-sided headache, associated with nausea, vomiting, and photophobia. His physical examination was unremarkable, and no laboratory studies were performed at that time. The patient was treated with intravenous ketorolac, diphenhydramine, and metoclopramide for symptomatic relief and discharged with follow-up instructions. A non-contrast CT scan was declined by the patient.

One week later, due to the persistence of his migraine, the patient presented to a neurologist now accompanied by disorientation, aphasia, dysarthria, and right-sided weakness. Given the acute nature of these new symptoms, a central nervous system infection or stroke was suspected, prompting a return visit to the ED.

Upon re-presentation, the patient was noted to be jaundiced and exhibited left upper extremity hemiplegia, dysarthria, and expressive aphasia. Initial laboratory studies as shown in Table 1 revealed significant hematologic abnormalities, including hemoglobin of 7.7 g/dL (normal: 14-17 g/dL), a platelet count of 27 x 10^9/L (normal: 150-450 x 10^9/L), elevated lactate dehydrogenase (LDH) of 852 U/L (normal: 135-225 U/L), and total bilirubin of 2.9 mg/dL (normal: 0-1.1 mg/dL).

**Table 1:** Blood laboratory results on presentation and discharge WBC: white blood cells; LDH: lactate dehydrogenase

Test	Initial Result	Final Result	Reference Range
WBC	10.6 x 10^9/L	8.7 x 10^9/L	4.5-11 x 10^9/L
Hemoglobin	7.7 g/dL	10 g/dL	14-17 g/dL
Hematocrit	23.3%	31.3%	39-50%
Platelets	27 x 10^9/L	305 x 10^9/L	150-450 x 10^9/L
LDH	852 U/L	165 U/L	135-225 U/L
Haptoglobin	73 mg/dL	130 mg/dL	30-200 mg/dL
Creatinine	1.14 mg/dL	0.87 mg/dL	0.60-1.3 mg/dL
Total Bilirubin	2.9 mg/dL	0.3 mg/dL	0-1.1 mg/dL
ADAMTS13 Activity	<5%	81%	>66.8%

A peripheral blood smear was done that revealed numerous schistocytes confirming MAHA. Neuroimaging including a non-contrast CT of the head, CT angiogram, and MRI brain without contrast was unremarkable for hemorrhage or stenosis, which is summarized in Table 2.

**Table 2:** Summary and interpretation of neuroimaging

Test	Findings	Interpretation
Non-contrast CT Head	No evidence of hemorrhage, ischemia, or structural abnormalities.	Ruling out intracranial hemorrhage and acute stroke.
CT Angiogram Head & Neck	No evidence of vascular abnormalities (e.g., vasculitis, arterial dissection, or cerebral venous sinus thrombosis (CVST)).	Exclusion of conditions like reversible cerebral vasoconstriction syndrome (RCVS) and CVST.
MRI Brain Without Contrast	No areas of restricted diffusion (no acute infarction), no intracranial hemorrhage, no intracranial mass. Tiny T2 hyperintensity in the right cerebellar hemisphere, likely representing a perivascular space. No midline shift, no mass effect, and normal ventricles and sulci. No cerebellar tonsillar herniation.	Ruling out stroke, hemorrhage, mass lesions, and intracranial pressure elevation. Perivascular space is a benign finding.

He was then taken to the ICU and started on emergent plasmapheresis along with a seven-day course of intravenous methylprednisolone. Empiric antimicrobial therapy was started with acyclovir, ceftriaxone, vancomycin, doxycycline, and dapsone. Due to marked thrombocytopenia, lumbar puncture was not done. The patient's subsequent ADAMST13 activity turned out to be less than 5%, and his Bethesda titer was calculated at 2.4 Bethesda units (BU). Given the severity of his presentation, caplacizumab was considered early in his course but was not given due to concerns about worsening thrombocytopenia. He improved quickly on plasmapheresis and steroids with rituximab added soon after. He was given a total of six sessions of plasmapheresis with significant improvement after the first several treatments. He was discharged after nine days with the resolution of all neurological deficits with normalization of LDH, haptoglobin, and bilirubin as shown in Table 1.

## Discussion

Neurological symptoms in TTP can range from mild to most severe but presentations vary widely. Hematologic abnormalities typically precede neurologic deficits, but as was the case in our patient, neurological symptoms can be the first manifestations of the disease [3,7]. At the time of the first visit, the patient did not have jaundice or mucocutaneous bleeding to suspect hematologic derangements. However, admittedly, subtle abnormalities could've been present at his initial ED visit. Persistence of symptoms and development of hemiplegia, aphasia, and dysarthria prompted further investigation in this case.

Stroke, reversible cerebral vasoconstriction syndrome (RCVS), cerebral venous sinus thrombosis (CVST), and posterior reversible encephalopathy syndrome (PRES) are among the differential diagnoses of the neurologic deficits seen in TTP. Given that no evidence of vasoconstriction could be seen from the imaging studies, RCVS was ruled out. Similarly, imaging studies failed to reveal any evidence of venous thrombosis, so one could also exclude CVST. There was no parieto-occipital white matter edema; therefore, PRES was excluded. A summary of imaging is presented in Table 2.

There are many case reports on TTP that present mainly with neurological features. One series focused on those patients whose major symptoms of altered mental status and seizures led to diagnosis without typical hematologic features at the time of presentation [5]. Another referred to one presenting with focal neurological deficits and headache, in whom initial neuroimaging indicated a stroke [6]. This, thus, calls for a high index of suspicion, especially in younger patients without any history of stroke or neurological disorders. With the normal early imaging findings, the presence of schistocytes and abnormal hematologic findings pointed to the diagnosis of TTP.

Low ADAMTS13 activity (<5%) confirmed the laboratory diagnosis in this case. The enzyme responsible for cleaving von Willebrand factor (vWF) multimers is ADAMTS13. Deficiency results in the accumulation of ultra-large vWF that induces platelet aggregation and microvascular thrombosis [8,6]. Activity assays for ADAMTS13 are very useful, but these tests are not widely available, and the results can be slow to appear. Thus, initiation of high-dose steroid treatment combined with plasmapheresis must be initiated while awaiting confirmation of the level of ADAMTS13 activity [9].

Previously, TTP carried a mortality rate of up to 90%. With technical advancements in modern diagnostic tools and newer therapies such as plasmapheresis and rituximab, mortality declined to approximately 10-20% [8]. Plasmapheresis filters the aberrant vWF multimers and restores ADAMTS13 levels while rituximab targets the autoimmune component underlying TTP [6]. Caplacizumab, a monoclonal antibody that blocks the interaction between vWF and platelets, should be considered in severe cases or those not responding to initial treatments [10]. Given the disruption to the interaction between the vWF and platelets causes an increased risk of bleeding this contributed to the decision to defer this modality especially given the drastic improvement clinically with the plasma exchanges. Long-term follow-up is necessary for detecting recurrence, which most frequently occurs in the first one to two years after diagnosis. Regular clinical assessments and laboratory tests should track ADAMTS13 activity, platelet count, and hemolysis markers such as LDH and bilirubin. Some sources state that rituximab may reduce recurrence risk and hence is an important drug in the long-term management of the disease [11,12]. This case illustrates the need for a high index of suspicion by clinicians for TTP in the presence of unexplained neurological symptoms.

## Conclusions

TTP is a rare and potentially fatal disorder that may present with atypical symptoms, such as a first-time complex migraine. A thorough workup, including a complete blood count, should be considered in patients presenting with new severe headaches to help identify secondary causes like TTP. Prompt treatment with plasmapheresis and steroids is essential, and caplacizumab should be considered in severe cases. Immunomodulatory therapies, such as rituximab, can help prevent relapses in refractory cases. This case also underscores the importance of multidisciplinary collaboration, as timely identification and treatment of TTP can significantly reduce morbidity and mortality.
